# The association between HIV and atherosclerotic cardiovascular disease in sub-Saharan Africa: a systematic review

**DOI:** 10.1186/s12889-017-4940-1

**Published:** 2017-12-15

**Authors:** Emily P. Hyle, Bongani M. Mayosi, Keren Middelkoop, Mosepele Mosepele, Emily B. Martey, Rochelle P. Walensky, Linda-Gail Bekker, Virginia A. Triant

**Affiliations:** 1 Medical Practice Evaluation Center, Massachusetts General Hospital, 50 Staniford St., 9th Floor, Boston, MA 02114-2696 USA; 2 Division of Infectious Diseases, Massachusetts General Hospital, Boston, MA USA; 3 Harvard Medical School, Boston, MA USA; 4 Cardiac Clinic, Department of Medicine, Groote Schuur Hospital and University of Cape Town, Cape Town, South Africa; 5 The Desmond Tutu HIV Centre, University of Cape Town, Cape Town, South Africa; 6 Department of Internal Medicine, Faculty of Medicine, University of Botswana, Gaborone, Botswana; 7 Botswana-Harvard AIDS Partnership, Gaborone, Botswana; 8 Harvard University Center for AIDS Research (CFAR), Boston, MA USA; 9 Division of Infectious Diseases, Brigham and Women’s Hospital, Boston, MA USA; 10 Division of General Internal Medicine, Massachusetts General Hospital, Boston, MA USA

**Keywords:** HIV, CVD, Atherosclerosis, Africa, Review

## Abstract

**Background:**

Sub-Saharan Africa (SSA) has confronted decades of the HIV epidemic with substantial improvements in access to life-saving antiretroviral therapy (ART). Now, with improved survival, people living with HIV (PLWH) are at increased risk for non-communicable diseases (NCDs), including atherosclerotic cardiovascular disease (CVD). We assessed the existing literature regarding the association of CVD outcomes and HIV in SSA.

**Methods:**

We used the PRISMA guidelines to perform a systematic review of the published literature regarding the association of CVD and HIV in SSA with a focus on CVD surrogate and clinical outcomes in PLWH.

**Results:**

From January 2000 until March 2017, 31 articles were published regarding CVD outcomes among PLWH in SSA. Data from surrogate CVD outcomes (*n* = 13) suggest an increased risk of CVD events among PLWH in SSA. Although acute coronary syndrome is reported infrequently in SSA among PLWH, limited data from five studies suggest extensive thrombus and hypercoagulability as contributing factors. Additional studies suggest an increased risk of stroke among PLWH (*n* = 13); however, most data are from immunosuppressed ART-naïve PLWH and thus are potentially confounded by the possibility of central nervous system infections.

**Conclusions:**

Given ongoing gaps in our current understanding of CVD and other NCDs in PLWH in SSA, it is imperative to ascertain the burden of CVD outcomes, and to examine strategies for intervention and best practices to enhance the health of this vulnerable population.

**Electronic supplementary material:**

The online version of this article (10.1186/s12889-017-4940-1) contains supplementary material, which is available to authorized users.

## Background

With more than 26 million people living with human immunodeficiency virus (PLWH) in sub-Saharan Africa (SSA), the daunting immediacy of health needs has necessitated expanding infrastructure to provide care for HIV infection [1]. As the beneficial effects of antiretroviral therapy (ART) are increasingly apparent [2], attention has now shifted to expanding this growing healthcare infrastructure to also encompass chronic care for non-infectious, highly prevalent co-morbidities [3, 4].

Non-communicable diseases (NCDs), specifically cardiovascular disease (CVD), increasingly affect the general population in SSA [5–7]. The effects of urbanization and increased life expectancy have been linked to an increased prevalence of traditional CVD risk factors, including changes in diet and exercise patterns [8], although CVD mortality has decreased in SSA over the past few decades [9, 10]. Concerns regarding the potential impact of CVD in SSA have focused appropriate and necessary attention on its diagnosis, treatment, and prevention [11, 12], and disparities in CVD prevention and treatment across SSA have been highly publicized [12–14]. Interventions in prevention, screening, and treatment have been shown to be effective and cost-effective among the general population [15, 16].

PLWH are at increased risk for CVD [17, 18], and a synergistic intersection of these two distinct epidemics may emerge in SSA [11, 19]. As access to ART expands, more PLWH are living past 50 years of age [20, 21] and face an increased risk of CVD due to traditional CVD risk factors alone [22]. The additional impact of HIV infection with its associated inflammation and prothrombotic state may further increase CVD risk. Limited data are available regarding optimal methods for CVD risk factor screening, primary and secondary prevention, risk stratification, outcomes, and management in PLWH living in SSA [23, 24]. Now is the time to anticipate and consider this challenge in the face of rising multimorbidity due to NCDs and chronic infectious diseases [25].

To complement previously published reviews of CVD among PLWH in SSA [26–33], we provide a systematic review and qualitative summary of the existing primary literature regarding the association of CVD and HIV in SSA with a focused emphasis on CVD surrogate and clinical outcomes. We identify current gaps in knowledge, focusing on the need for additional outcomes data, implementation strategies, and best practices for risk factor modification and treatment.

## Methods

### Search strategy

**Fig. 1:**
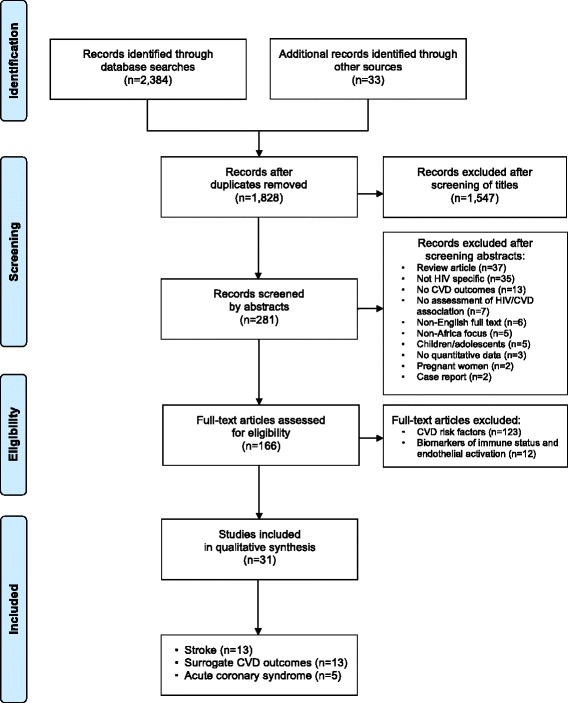
Flow diagram for the selection of studies. We followed PRISMA guidelines for screening articles in the systematic review. We identified articles via a systematic search of the PubMed database and derived additional records from reference lists of previously identified articles or co-author input. Articles were screened first by title, then by abstract, and finally on explicit inclusion/exclusion criteria. Full-text articles considered eligible for inclusion were categorized based on emphasis of CVD risk factors, biomarkers of immune status and endothelial activation, surrogate CVD outcomes, acute coronary syndrome, and stroke

This systematic review of CVD surrogate and clinical outcomes in PLWH in SSA was conducted according to Preferred Reporting Items for Systematic Reviews and Meta-Analyses (PRISMA) guidelines (Fig. 1) [34]. We identified articles published from January 2000 to March 2017 through searches in PubMed that included Medical Subject Headings (MeSH) terms, “Africa” and “HIV,” as well as any of the following terms: “cardiovascular disease,” “cardiometabolic,” “metabolic syndrome,” “myocardial infarction,” “acute coronary syndrome,” “dyslipidemia,” “diabetes,” “dysglycemia,” “hypertension,” “surrogate marker,” “cIMT,” “pulse wave velocity,” “aortic augmentation index,” “ankle-brachial index,” “endothelial activation,” “radial tonometry,” “flow-mediated dilation,” or “stroke.” We included additional articles found in review of bibliographies or suggested by co-authors based on their relevance to the selected search terms. The full list of search terms is reported in Additional file 1.

### Study selection

We selected relevant articles in a stepwise manner. Two co-authors (EPH and EBM) independently screened articles by title to ensure that the analysis included articles regarding PLWH, CVD, and sub-Saharan Africa. We next reviewed the selected abstracts, using the following inclusion criteria: study population without children or pregnant women; primary quantitative data; specific CVD risk factors or outcomes; assessment of an association between CVD and HIV; English written text. Last, we assessed the remaining full-text articles for eligibility and determined which studies would be included in qualitative synthesis.

### Data extraction

Two co-authors (EPH and MM) extracted data for qualitative synthesis, including location, year of study, study design, sample size, population age (in years), and ART status, and summarized the main findings of eligible analyses. We did not perform quantitative synthesis because published study data included diverse populations and study designs, rarely offered effect sizes, and often included unmeasured confounders.

## Results

### Study selection

**Fig. 2:**
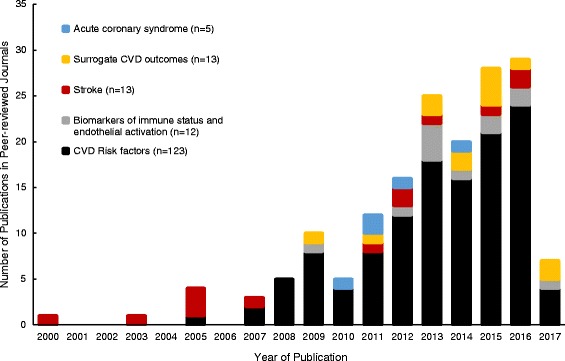
Publication year for studies on HIV and CVD risk factors and outcomes in sub-Saharan Africa. Full-text articles were stratified by year of publication and description of CVD risk factors, biomarkers of immune status and endothelial activation, surrogate CVD outcomes, acute coronary syndrome, or stroke. Of the 166 studies assessed in this review, the majority (*n* = 102) were published between 2013 and 2016. Only 3 months of 2017 publications were included

Full-text articles selected for eligibility were further separated into CVD risk factors (*n* = 123), biomarkers of immune status and endothelial activation (*n* = 12), and CVD outcomes (*n* = 31) (Fig. 2). Studies categorized as CVD risk factors or biomarkers of immune status and endothelial activation are reported in an additional reference list (see Additional file 2). The eligible 31 analyses evaluated the impact of HIV status on surrogate CVD outcomes (*n* = 13), acute coronary syndrome (ACS) (*n* = 5), and stroke (*n* = 13) for inclusion in qualitative synthesis and detailed data extraction.

### CVD risk factors

Traditional CVD risk factors are prevalent in SSA among the general population and include hypertension (6-22%) [35–37], dyslipidemia (5–70%) [38], diabetes (1-12%) [35, 39], and smoking (males 15%, females 0.6%) [40, 41]. Despite these high prevalence estimates, almost 67% of diabetics and 50% of hypertensives are thought to be unaware of their status [14, 42]. Tobacco use varies widely in different regions and is likely under-reported, particularly among women [40].

Among PLWH in the US and Europe, traditional CVD risk factors are highly prevalent, even when accounting for age [43]. Hypertension is associated with CVD risk in PLWH [44], and an important interplay also occurs between lipid indices, HIV, chronic inflammation, and antiretroviral medications [45–47]. PLWH are at risk for impaired glucose tolerance (IGT) and for diabetes [48]. Smoking is at least twice as prevalent in PLWH in the US [49] and has a greater impact on overall mortality than HIV itself in the setting of available ART [50].

Considerably less is known about the determinants and impact of CVD risk factors among PLWH in SSA. The association of hypertension with higher CD4 counts and older age suggests that hypertension may be a substantial problem among PLWH in SSA as they age on ART [22, 51–54]. Wide ranges in prevalence of IGT (16-24%) [55–58] and diabetes (1-18%) [58–64] are reported in PLWH in SSA. Total cholesterol, low-density lipoprotein (LDL), and high-density lipoprotein (HDL) all rise following ART initiation, yet a relatively smaller increase in HDL results in a pro-atherosclerosis lipid profile [65–71]. Smoking is a public health concern in SSA, yet the role of HIV with regards to smoking rates in this setting remains unclear. Some cohorts demonstrate an increased prevalence of smoking compared with the general public in the region (23% vs 16%) [72, 73], while others show decreased smoking rates among PLWH (48% vs 31%) [74] or no difference [69, 75–78].

Chronic inflammation plays a role in the development of atherosclerotic CVD among PLWH, and evidence is mounting for the contribution of viremia and immunosuppression towards the premature development of CVD [79, 80]. Although several SSA studies demonstrated immune activation among both ART-naïve and –experienced PLWH [78, 81–84], a South African study focused on older PLWH (≥50 years) demonstrated a reduction in inflammatory markers (i.e., CRP, IL-1, IL-6, and TNF-α) after ART-initiation [85]. Evaluating the role of immune activation and cardiovascular risk in a cohort of PLWH on ART in southwestern Uganda, lower absolute levels of sCD14 and IL-6, markers of monocyte activation and generalized inflammation, were found to be significantly associated with lower future carotid intima media thickness (cIMT), a marker of preclinical atherosclerosis, after adjusting for traditional CVD risk factors [86]. Several studies in SSA have also consistently demonstrated that PLWH experience excess endothelial activation [78, 87], which persists even after the introduction of ART [82]. Given the potential impact of chronic inflammation on PLWH in SSA and possible concomitant mediators of inflammation from other infections endemic to the region [88], further research into the pathogenesis of atherosclerotic CVD among PLWH in SSA will be valuable and may offer additional insights into molecular markers, predictive tests, and treatment options.

Patterns of screening and diagnosis of CVD risk factors in SSA may differ by HIV status. PLWH are less likely to be asked about CVD risk factors at routine clinical visits compared with HIV-uninfected groups [89]. However, when CVD risk factors are actively assessed, PLWH on ART may be more likely to be diagnosed with hypertension or diabetes than patients with unknown HIV status. In a South African study using pharmacy records, for instance, hypertension was the most common second diagnosis among PLWH on ART, making it more common than tuberculosis (TB) [25].

PLWH may also be more likely to have multiple CVD risk factors, thus increasing their overall risk of CVD outcomes. Among patients on ART for more than 34 months in Cameroon, 61% had one CVD risk factor, and 18% had two or more CVD risk factors [90]. Among virologically suppressed PLWH ages 40-50 in Botswana, the American Heart Association (AHA)/American College of Cardiology (ACC) risk equation demonstrated elevated risk of CVD outcomes, which correlated with cIMT measurements [91]. However, only 3.6% of patients who were actively screened for CVD risk factors in a South African study were found to have more than a 10% risk of a CVD event in the next 10 years, using a risk stratification tool from the World Health Organization and International Society of Hypertension [63]. Strategies to estimate CVD risk among PLWH have not been formally assessed in SSA, and accurate risk prediction algorithms will be important to guide preventive care.

### CVD outcomes

Although data are increasingly available regarding CVD risk factors among PLWH in SSA, data on CVD outcomes remain scarce (Fig. 2).

#### Surrogate CVD outcomes

Assessment of surrogate CVD outcomes (e.g., cIMT, pulse wave velocity (PWV), aortic augmentation index (AI), ankle-brachial index (ABI), flow-mediated dilation (FMD), radial tonometry) has demonstrated the potential for an increased risk of CVD events among PLWH in SSA (Table 1). Although the surrogate outcomes studied are diverse and reflect discrete vascular functions, the majority of studies showed increased atherosclerosis among PLWH, either versus controls or in relation to HIV disease duration or treatment status. Although some studies demonstrate no evidence for increased atherosclerosis among PLWH [78, 92], most studies show early subclinical atherosclerosis by surrogate CVD outcomes [81, 93–98]. A large Ugandan study demonstrated subclinical atherosclerosis via cIMT in 18% of PLWH attending clinic with higher risk among ART-experienced or older patients with elevated body mass index (BMI) or LDL [95]. A second study in Uganda diagnosed arterial stiffness by ABI in 33% (19%) of male (female) PLWH on ART for 7 years, which was twice the prevalence compared to age- and sex-matched HIV-uninfected controls after adjustment for traditional CVD risk factors [97]. A cross-sectional study from South Africa demonstrated that 12% of PLWH (median age 41 years, 69% female) had subclinical atherosclerosis by cIMT that was associated with traditional but not HIV-related factors [99]. Studies comparing surrogate CVD outcomes in PLWH versus comparator HIV-uninfected groups will further elucidate the role that HIV could play in the development of atherosclerosis. The long-term implications of these altered vascular indices remain unknown among PLWH in SSA and warrant further investigation.

**Table 1:** Published studies on surrogate CVD outcomes in PLWH in sub-Saharan Africa

Study (location, dates)	Study Design	N =	Age (years)^a^	ART-status	Findings
Fourie et al. [93] (SA, 2005)	Case-control	PLWH: 300 HIV-: 300	44 ± 8 44 ± 8	100% ART-naïve	• Untreated HIV associated with higher biomarkers of endothelial injury
Lazar et al. [92] (Rwanda, 2005)	Prospective cohort	PLWH: 276 HIV-: 67	35 ± 7 41 ± 10	59% ART-naïve 41% on ART	• HIV not associated with increased arterial wave reflection
Botha et al. [132] (SA, 2005-10)	Prospective cohort	PLWH: 137	no ART: 47.6 ± 1.9 on ART: 47.8 ± 1.6	52% ART-naïve 48% on ART	• ART exposure associated with higher pulse pressure
Fourie et al. [78] (SA, 2005-10)	Prospective cohort	PLWH: 144 HIV-: 165	no ART: 48 ± 1 on ART: 49 ± 1 50 ± 1	54% ART-naïve 46% on ART	• >5y HIV infection associated with increased biomarkers of endothelial activation
Ngatchou et al. [94] (Cameroon, 2009-10)	Cross-sectional	PLWH: 108 HIV-: 96	39 ± 11 41 ± 12	100% ART-naïve	• HIV associated with aortic stiffness as assessed by radial tonometry
Ngatchou et al. [133] (Cameroon, 2009-10)	Cross-sectional	PLWH: 238	no ART: 39 ± 11 on ART: 41 ± 12	45% ART-naïve 55% on ART	• ART exposure associated with increased pulse pressure and augmentation index among ART-experienced patients compared to untreated PLWH
Ssinabulya et al. [95] (Uganda, 2012)	Cross-sectional	PLWH: 245	37 (31-37)	59% ART-naïve 41% on ART	• HIV associated with 18% risk of pre-clinical carotid atherosclerosis on ultrasound imaging
Awotedu et al. [96] (SA, 2012-13)	Cross-sectional	PLWH: 106 HIV-: 63	no ART: 36 ± 11 on ART: 40 ± 10 36 ± 11	51% ART-naïve 49% on ART	• HIV associated with increased aortic pulse wave velocity • Highest increase among ART-experienced
Schoffelen et al. [99] (SA, 2013)	Cross-sectional	PLWH: 904	41 (35-48)	13% ART-naïve 87% on ART	• cIMT associated with traditional CVD risk factors, not HIV-specific factors
Siedner et al. [97] (Uganda, 2013-14)	Cross-sectional	PLWH: 105 HIV-: 100	49 (45-51) 50 (46-54)	100% on ART	• HIV associated with twice the risk of arterial stiffness as assessed by calculating ankle-brachial index
Feinstein et al. [98] (Uganda, not stated)	Cross-sectional	PLWH: 105 HIV-: 100	49 ± 6 52 ± 9	100% on ART	• HIV not associated with carotid intima media thickness
Gleason et al. [81] (Ethiopia, not stated)	Cross-sectional	PLWH: 281 HIV-: 36	no ART: 38 (32-45) EFV: 38 (32-45) LPV/r: 39 (35-44) NVP: 37 (32-42) 39 (29-45)	18% ART-naïve 82% on ART	• Use of EFV & LPV/r, nut not NVP, is associated with elevated pulse wave velocity, normalized cIMT, and abnormal FMD
Mosepele et al. [91] (Botswana, not stated)	Cross-sectional	PLWH: 208	39 (5)	25% ART-naïve 75% on ART	• Atherosclerotic CVD risk score and cIMT measurement similarly identify high CVD risk

^a^mean ± SD or median (IQR)

*PLWH* people living with HIV, *SA* South Africa, *ART* antiretroviral therapy, *EFV* efavirenz, *LPV/r* lopinavir/r, *NVP* nevirapine, *cIMT* carotid intima-media thickness, *FMD* flow-mediated dilation

#### Acute coronary syndrome

Acute coronary syndrome (ACS) is reported relatively infrequently in SSA [11, 27]. Events may be underreported due to “silent” myocardial infarctions, limited access to diagnostic tests, and lack of patient and healthcare provider awareness. However, a recent study shows that more than 5% of cardiac hospitalizations are attributable to ACS in South Africa [100]. With increasing life expectancy and a growing burden of CVD risk factors, the incidence of ACS and subsequent morbidities may rise in SSA.

Specifically among PLWH living in SSA, ACS is not reported frequently, and no studies have investigated ACS incidence in PLWH relative to control patients [24, 27]. In a large cohort study in Soweto, South Africa following 5328 new cases of heart disease, ACS was described in only 3% of the 518 cases of cardiac hospitalization among PLWH (although HIV testing was performed only when “clinically indicated”) (Table 2) [24]. The presentation of ACS was further examined in a separate study from the same population in which all patients with ACS were tested for HIV. In comparison to HIV-uninfected controls, 30 HIV-infected cases were younger and more likely to have a history of smoking but less likely to have traditional CVD risk factors such as diabetes, hypertension, and dyslipidemia [101]. Angiographic features among the HIV-infected cases revealed acute thrombus with a low burden of underlying atherosclerotic plaque, consistent with US studies showing fewer involved vessels and a greater relative burden of inflammatory plaque [102]. A follow-up study of this same study population demonstrated a greater prevalence of coagulopathy among the PLWH who presented with myocardial infarction, specifically elevated protein C levels [103]; anti-phospholipid antibodies were not found to be associated with ACS among PLWH [104].

**Table 2:** Published studies on acute coronary syndrome in PLWH in sub-Saharan Africa

Study (location, dates)	Study Design	N =	Age (years)^a^	ART-status	Findings
Becker et al. [101] (SA, 2004-08)	Prospective case-control	ACS + PLWH: 30 ACS + HIV-: 30	43 ± 7 54 ± 13	100% ART-naïve	• Traditional risk factors more prevalent in HIV-, except for smoking • PLWH more likely to have single vessel disease and greater thrombus burden • PLWH more likely to have MACE and need TLR at follow-up
Becker et al. [103] (SA, 2004-08)	Same study population as above				• PLWH with ACS more likely to have lower protein C and higher Factor VIII, Anti-cardiolipin IgG and Anti-prothrombin IgG
Becker et al. [104] (SA, 2004-08)	Prospective case-control	ACS-PLWH: 30 ACS + PLWH: 30 ACS + HIV-: 30	41 ± 8 43 ± 7 54 ± 13	100% ART-naïve	• PLWH are more likely to have anti-phospholipid antibodies but this is not associated with ACS
Sliwa et al. [24] (SA, 2006-08)	Cohort	PLWH: 518	39 ± 13	46% ART-naïve 54% on ART	• 170 (32.8%) were new HIV diagnoses • 14 (2.7%) were admitted with ACS and 18 (3.5%) with cerebrovascular disease
Redman et al. [105] (SA, 2008-11)	Prospective cohort of vascular surgery patients	PLWH: 73 HIV-: 152	41 ± 10 56 ± 13	68% ART-naïve 23% on ART	• Lower RCRI score among PLWH • No difference in 30 day outcomes (13% vs 15%), even though PLWH were younger

^a^mean ± SD or median (IQR)

*PLWH* people living with HIV, *SA* South Africa, *ACS* acute coronary syndrome, *ART* antiretroviral therapy, *MACE* major adverse cardiovascular events, *TLR* target lesion revascularization, *RCRI* Revised Cardiac Risk Index

PLWH may be at higher risk for coronary artery disease than suggested by traditional CVD risk factors alone, given data on post-operative cardiovascular morbidity and mortality. In a South African cohort of 255 patients undergoing vascular surgery, 32% were HIV-infected of whom 23% were on ART [105]. When compared to vascular surgery patients confirmed to be HIV-uninfected, PLWH were equally as likely to have myocardial infarction as measured by troponin elevations on post-operative day three or to experience 30-day mortality. PLWH were less likely to have known traditional CVD risk factors and were less likely to be on cardiovascular medications; it is not known if the prevalence of traditional CVD risk factors was lower among PLWH or if they were less likely to have been previously screened for these diseases.

#### Stroke

Stroke is a substantial problem in the general population in SSA and is associated with high rates of mortality, morbidity, and post-stroke disability [13, 106–108]. An association between HIV infection and stroke has been described in high-income countries [109–111]; data emerging from SSA also suggest a high prevalence of stroke among PLWH. Relative to ACS, data regarding stroke among PLWH in SSA are more extensive, which may be due to the more readily recognizable symptoms of stroke that are likely to result in presentation to medical attention, as well as the high prevalence of hypertension in SSA. Three distinct study designs have been used to investigate an association of stroke and HIV in SSA: 1) comparisons of PLWH and HIV-uninfected patients who present with stroke; 2) comparisons of stroke patients with population controls, evaluating for HIV status in both groups 3) assessment of cohorts of PLWH, some of whom have had stroke (Table 3).

**Table 3:** Published studies on stroke in PLWH in sub-Saharan Africa

Study (location, dates)	Study Design	N =	Age (years)^a^	ART-status	Findings
Study population: stroke patients					
Patel et al. [116] (SA, 1987-2002)	Retrospective case-control^b^	PLWH: 56 HIV-: 154	15-44	100% ART-naïve	• No significant differences between PLWH and HIV- patients with stroke regarding cardiac etiologies or angiography
Hoffmann et al. [117] (SA, 1992-98)	Prospective case-control^b^	PLWH: 22 HIV-: 23	29.1 (20-42) 31.0 (19-44)	100% ART-naïve	• PLWH with fewer traditional CVD risk factors • 20 of 22 (91%) PLWH with unknown etiology for stroke • All PLWH were diagnosed with HIV at admission for stroke
Mochan et al. [114] (SA, 1999-2000)	Case series of PLWH and stroke	PLWH: 35	32.1 (20-61)	100% ART-naïve	• 94% ischemic; 6% hemorrhagic • LP showed infection in 10 of 33 PLWH • 17 PLWH had coagulopathy
Tipping et al. [113] (SA, 2000-06)	Prospective cohort of stroke patients	PLWH: 67 HIV-: 1020 *(all subjects)* PLWH: 61 HIV-: 205 *(<46y)*	33.4 (19-76) 64 (17-96)	12% < 6 months ART 88% ART-naïve	• 96% ischemic; 4% hemorrhagic • ID etiologies predominant among PLWH (37% vs 8%) • 20% of PLWH had HIV vasculopathy • Fewer traditional risk factors among PLWH <46 years
Kumwenda et al. [112] (Malawi, 2001-02)	Prospective case-control	PLWH: 47 HIV-: 51	37.5 ± 13.1 58.6 ± 16.8	100% ART-naïve	• 11 (23%) of PLWH diagnosed with infectious etiologies of stroke
Heikinheimo et al. [115] (Malawi, 2008-09)	Cohort of 1st time stroke	PLWH: 50 HIV-: 84	39.8 ± 12.4 61.9 ± 14.0	22% on ART	• No difference in outcomes between PLWH and HIV- • PLWH were younger with fewer CVD risk factors
Owolabi et al. [37] (Nigeria, 2008-10)	Prospective cohort of stroke patients (18-40 years)	PLWH: 6 HIV-: 65	31.9 ± 6	Not stated	• 8.5% were PLWH but not all patients were tested • HIV is 5th most common risk factor
Study population: PLWH					
Longo-Mbenza et al. [134] (DRC, 2004-08)	Cross-sectional	PLWH: 116	45.3 ± 8.5 (men) 42.5 ± 11.2 (women)	100% of stroke patients on ART	• 17 (15%) PLWH had stroke • 94% ischemic; 6% ICH • Uncertain ID workup • Fewer traditional risk factors among stroke patients, but lower CD4 and more WHO Stage 3-4
Divala et al. [135] (Malawi, 2014)	Cross-sectional	PLWH: 952	43.0 ± 10.2	4.1% ART-naïve 95.9% on ART	• Self-reported past stroke: 4.3%
Study population: stroke (cases) and non-stroke from community (controls)					
Walker et al. [118] (Tanzania, 2003-06)	Case-control^b^	Stroke: 201 (PLWH: 25) Controls: 398 (PLWH: 15)	61.7 (15.0) and 68.8 (14.8)^c^ 61.4 (13.1) and 69.4 (14.6)^c^	100% ART-naïve	• Stroke associated with traditional risk factors • HIV independently associated with stroke (aOR, 5.61)
Benjamin et al. [136] (Malawi, 2011-12)	Case-control^d^	Stroke: 222 (PLWH: 69) Controls: 503 (PLWH: 95)	60 (42-70) 57 (42-67)	17% ART-naïve 7% ART <6 mo 6% ART ≥6 mo 9% ART-naïve 1% ART <6 mo 8% ART ≥6 mo	• 78% ischemic; 22% hemorrhagic • Stroke associated with HIV (aOR, 3.28), especially if ART started in past 6 months (aOR, 15.6) • No effect modification for HIV and HTN • Diabetes and smoking were independently associated
Asiki et al. [137] (Uganda, not stated)	Retrospective case-control^b^	Stroke: 31 (PLWH: 5) Controls: 135 (PLWH: 8)	59.0 (13.7) 60.2 (13.7)	62% of PLWH are ART-naïve	• Increased risk of stroke among HIV+ (16% vs 6%) • Potentially biased sample given patients included only if they had frozen specimen available for varicella IgG testing
Mochan et al. [138] (SA, not stated)	Case-control^e^	Stroke: 33 (PLWH: 33) Controls: 66 (PLWH: 33)	32.1 (20-61)	Not stated	• Protein S deficiency associated with HIV, not stroke

^a^mean ± SD or median (IQR)

^b^matched by age/sex

^c^two study regions (Dar-es-Salaam and Hai)

^d^matched by age, sex, socioeconomic status, season of admission

^e^matched by age, sex, and CD4

*PLWH* people living with HIV, *SA* South Africa, *ART* antiretroviral therapy, *LP* lumbar puncture, *ID* infectious disease, *DRC* Democratic Republic of Congo, *ICH* intracerebral hemorrhage, *WHO* World Health Organization, *aOR* adjusted odds ratio, *HTN* hypertension

HIV infection is frequently reported among patients enrolled in studies that assessed new stroke patients. Almost 3% of all inpatient admissions were due to stroke in a Malawi cohort comprising 70% PLWH [112]. A prospective cohort of young stroke patients (<46 years) admitted to a South African stroke referral hospital demonstrated HIV infection in 23%, more than twice the population’s HIV prevalence (11%) [113]. In a hospital-based South African cohort study of PLWH with stroke, 57% of patients presented with stroke as their initial presentation of HIV [114]. Although morbidity and mortality remain very high among patients after stroke in SSA, outcomes of PLWH versus the general population post-stroke did not differ substantially in terms of case-fatality rates or disabilities [112, 115].

When comparing stroke patients with and without HIV, some studies found no differences in risk factors or outcomes [116]. Other studies found that PLWH with stroke were more likely to be younger and have fewer traditional CVD risk factors than stroke patients without HIV [112, 113, 115, 117]. Such results suggest a possible role of HIV infection itself or other nontraditional risk factors in conferring stroke risk among PLWH in this setting. However, such studies have predominantly assessed patients who are ART-naïve and often recently diagnosed with HIV; these patients are more likely to be immunosuppressed and at increased risk for opportunistic infections which could impact the central nervous system. Infectious etiologies of stroke are more common in PLWH, and rigorous diagnostic workup in one study revealed infectious causes in 23% of PLWH compared to none of the HIV-uninfected [112]. Other studies found a similarly high probability of infectious etiology for stroke when comprehensive diagnostic testing was performed [113, 114].

Two studies that used a case-control study design to compare stroke patients with non-stroke controls from the community demonstrated an independent association between HIV and stroke, as well as an association between stroke and traditional CVD risk factors [118, 119]. In one study, all PLWH were ART-naïve [118]; in the other study, an association between the timing of ART initiation (within the past 6 months) and stroke was significant, suggesting a possible role for immune reconstitution syndrome [119]. Neither study outlined a thorough diagnostic workup for other infectious causes of stroke, which could be an important, unmeasured confounder.

Data regarding stroke in PLWH on ART for more than a year and therefore at reduced risk for infectious etiologies will be essential to extend our understanding of CVD in PLWH in SSA.

## Discussion

### Summary of literature and current knowledge gaps

Although our review of the published literature underscores the increasing availability of data on this topic (Fig. 2), substantial limitations in existing data remain. Published data still frequently include small sample sizes and are likely under-powered to detect differences. Additionally, the patient populations are frequently younger than 50 years and therefore do not reflect the aging of the epidemic, and many studies do not evaluate the impact of socio-economic status on CVD risk factors and outcomes. Many studies continue to present data on mixed populations of ART-naïve and ART-experienced patients so that the impact of immunosuppression and longitudinal treatment with ART cannot be assessed. Moreover, in case-control studies that include an HIV-uninfected control group, patients are rarely proactively tested for HIV, so the control group likely includes a mixed population of people living with and without HIV. Studies that include ART-naïve PLWH often do not include a thorough diagnostic workup for infectious etiologies of stroke. CVD outcomes, particularly ACS, peripheral arterial disease, and venous thromboembolic disease, are infrequently identified and reported, so the impact of any increasing prevalence of CVD risk factors remains uncertain. Most studies report cross-sectional data, which do not capture risks, exposures, and outcomes over time. Case-control studies often include unmeasured confounders, including infectious co-morbidities such as tuberculosis or syphilis. Last, data are often from clinical cohorts or trials, which might not be representative of PLWH engaged in care in large public health programs.

Larger cohort studies among PLWH and HIV-uninfected patients in SSA during the ART era will clarify the incidence, prevalence, and attributable morbidity and mortality of CVD risk factors and outcomes in these populations. Focused investigation regarding possible interactions of traditional CVD risk factors with HIV infection, ART, and chronic inflammation can elucidate potential pathophysiologic mechanisms for further comparison with data from the US and Europe to guide prevention and management strategies. Innovative models of integrated care, which are appropriate and scalable for enhanced CVD risk management, should be extensively studied in SSA. Such models should leverage successful HIV programs, where feasible.

Prospective ascertainment of best practices for CVD risk factor screening and CVD management in SSA will identify future interventions for PLWH. Abundant opportunities for CVD risk factor screening exist for PLWH given frequent interactions with community testing campaigns and clinical care [120, 121], but subsequent linkage to and retention in clinical care after diagnosis is essential in order to optimize both HIV and CVD outcomes [36]. The identification of optimal ART, anti-hypertensive, lipid, and diabetes management strategies for PLWH is needed, as well as implementation of effective methods of lifestyle modification, nutrition education, smoking cessation, and expansion of opportunities to exercise. The Randomized Trial to Prevent Vascular Events in HIV (REPRIEVE) trial, with its sites in SSA, will provide new data on CVD risk reduction in this setting [122].

### Policy implications

As more PLWH are successfully linked to clinical care and treated with ART, attention must turn to maintaining their restored health in order to realize the full health benefits of ART. The existing and expanding health care infrastructure developed for HIV presents an opportunity to incorporate additional preventative interventions for chronic disease complications to decrease morbidity and improve quality of life for PLWH.

Some argue that integration of non-HIV medical services with HIV clinical care could decrease quality of care and dilute the effectiveness of current HIV programs. Concerns that investment in HIV care has detracted from investments in other forms of health care, such as immunizations [123], have tempered enthusiasm for expanding HIV health systems to include additional health care directives.

However, the potential benefits of an integrated approach to medical care for PLWH in SSA and other resource-limited settings could be profound, and support for this approach has been fast-growing [20, 124–126]. The established and expanding HIV infrastructure could be an ideal foundation on which to build additional interventions and management strategies for other chronic diseases [29, 127]. An emphasis on HIV as a chronic disease that requires life-long management has also influenced the paradigm of care [128–130] and encourages the use of current HIV infrastructure as a platform for management of chronic non-infectious co-morbidities. In regions where primary care services are already more established, existing clinical services could alternatively serve as a platform for decentralizing HIV care and integrating with existing NCD prevention and management efforts [131].

## Conclusions

The limited data available from PLWH in SSA regarding atherosclerotic CVD outcomes suggests an increased risk of early atherosclerosis and stroke. Given ongoing gaps in our current understanding of CVD in PLWH in SSA, now is the time to advance targeted research priorities and determine the burden of CVD outcomes, strategies for intervention, and best practices to enhance the health of this vulnerable population.

## Additional files

Additional file 1: Search Strategy. (PDF 10 kb)

Additional file 2: Additional References. The following references primarily focused on CVD risk factors (*n* = 123) and biomarkers of immune status and endothelial activation (*n* = 12). These publications were assessed for eligibility but were excluded from the final qualitative analysis. (PDF 63 kb)

## Abbreviations

ABI: Ankle-brachial index
ACC: American College of Cardiology
ACS: Acute coronary syndrome
AHA: American Heart Association
AI: Aortic augmentation index
ART: Antiretroviral therapy
BMI: Body mass index
cIMT: Carotid intima media thickness
CVD: Cardiovascular disease
FMD: Flow-mediated dilation
HDL: High-density lipoprotein
IGT: Impaired glucose tolerance
LDL: Low-density lipoprotein
NCD: Non-communicable disease
PLWH: People living with HIV
PRISMA: Preferred Reporting Items for Systematic Reviews and Meta-Analyses
PWV: Pulse wave velocity
SSA: Sub-Saharan Africa
