# Supplementary material for: The association between HIV and atherosclerotic cardiovascular disease in sub-Saharan Africa: a systematic review
Source: BMC Public Health. 2017 Dec 15;17:954. doi: 10.1186/s12889-017-4940-1 (PMC5732372; doi:10.1186/s12889-017-4940-1)
Supplement: Supplementary file 1 — Search Strategy. (PDF 10 kb) [file 12889_2017_4940_MOESM1_ESM.pdf]

ADDITIONAL FILE 1

Study Strategy

PubMed:

("cardiovascular system"[MeSH Terms] OR ("cardiovascular"[All Fields] AND "system"[All Fields]) OR "cardiovascular system"[All Fields] OR "cardiovascular"[All Fields]) OR ("heart"[MeSH Terms] OR "heart"[All Fields] OR "cardiac"[All Fields]) OR cardiometabolic[All Fields] AND ("hiv"[MeSH Terms] OR "hiv"[All Fields]) AND ("africa"[MeSH Terms] OR "africa"[All Fields])

(metabolic[All Fields] AND ("syndrome"[MeSH Terms] OR "syndrome"[All Fields])) AND ("hiv"[MeSH Terms] OR "hiv"[All Fields]) AND ("africa"[MeSH Terms] OR "africa"[All Fields])

("hiv"[MeSH Terms] OR "hiv"[All Fields]) AND ("myocardial infarction"[MeSH Terms] OR ("myocardial"[All Fields] AND "infarction"[All Fields]) OR "myocardial infarction"[All Fields]) OR (acute[All Fields] AND ("heart"[MeSH Terms] OR "heart"[All Fields] OR "coronary"[All Fields])) AND ("africa"[MeSH Terms] OR "africa"[All Fields])

("stroke"[MeSH Terms] OR "stroke"[All Fields]) AND ("hiv"[MeSH Terms] OR "hiv"[All Fields]) AND ("africa"[MeSH Terms] OR "africa"[All Fields])

("hypertension"[MeSH Terms] OR "hypertension"[All Fields]) AND ("hiv"[MeSH Terms] OR "hiv"[All Fields]) AND ("africa"[MeSH Terms] OR "africa"[All Fields])

("diabetes mellitus"[MeSH Terms] OR ("diabetes"[All Fields] AND "mellitus"[All Fields]) OR "diabetes mellitus"[All Fields] OR "diabetes"[All Fields] OR "diabetes insipidus"[MeSH Terms] OR ("diabetes"[All Fields] AND "insipidus"[All Fields]) OR "diabetes insipidus"[All Fields]) OR dysglycemia[All Fields] AND ("hiv"[MeSH Terms] OR "hiv"[All Fields]) AND ("africa"[MeSH Terms] OR "africa"[All Fields])

("dyslipidemias"[MeSH Terms] OR "dyslipidemias"[All Fields] OR "dyslipidemia"[All Fields]) AND ("hiv"[MeSH Terms] OR "hiv"[All Fields]) AND ("africa"[MeSH Terms] OR "africa"[All Fields])

(("endothelium"[MeSH Terms] OR "endothelium"[All Fields] OR "endothelial"[All Fields]) AND activation[All Fields]) AND ("hiv"[MeSH Terms] OR "hiv"[All Fields]) AND ("africa"[MeSH Terms] OR "africa"[All Fields])

("biomarkers"[MeSH Terms] OR "biomarkers"[All Fields] OR ("surrogate"[All Fields] AND "markers"[All Fields]) OR "surrogate markers"[All Fields]) AND ("cardiovascular diseases"[MeSH Terms] OR ("cardiovascular"[All Fields] AND "diseases"[All Fields]) OR "cardiovascular diseases"[All Fields] OR ("cardiovascular"[All Fields] AND "disease"[All Fields]) OR "cardiovascular disease"[All Fields]) AND ("hiv"[MeSH Terms] OR "hiv"[All Fields]) AND ("africa"[MeSH Terms] OR "africa"[All Fields])

("ankle brachial index"[MeSH Terms] OR ("ankle"[All Fields] AND "brachial"[All Fields] AND "index"[All Fields]) OR "ankle brachial index"[All Fields]) AND ("hiv"[MeSH Terms] OR "hiv"[All Fields]) AND ("africa"[MeSH Terms] OR "africa"[All Fields])

(("aorta"[MeSH Terms] OR "aorta"[All Fields] OR "aortic"[All Fields]) AND augmentation[All Fields] AND ("abstracting and indexing as topic"[MeSH Terms] OR ("abstracting"[All Fields] AND "indexing"[All Fields] AND "topic"[All Fields]) OR "abstracting and indexing as topic"[All Fields] OR "index"[All Fields])) AND ("hiv"[MeSH Terms] OR "hiv"[All Fields]) AND ("africa"[MeSH Terms] OR "africa"[All Fields])

("pulse wave analysis"[MeSH Terms] OR ("pulse"[All Fields] AND "wave"[All Fields] AND "analysis"[All Fields]) OR "pulse wave analysis"[All Fields] OR ("pulse"[All Fields] AND "wave"[All Fields] AND "velocity"[All Fields]) OR "pulse wave velocity"[All Fields]) AND ("hiv"[MeSH Terms] OR "hiv"[All Fields]) AND ("africa"[MeSH Terms] OR "africa"[All Fields])

cIMT[All Fields] AND ("hiv"[MeSH Terms] OR "hiv"[All Fields]) AND ("africa"[MeSH Terms] OR "africa"[All Fields])

(radial[All Fields] AND ("manometry"[MeSH Terms] OR "manometry"[All Fields] OR "tonometry"[All Fields])) AND ("hiv"[MeSH Terms] OR "hiv"[All Fields]) AND ("africa"[MeSH Terms] OR "africa"[All Fields])

(flow[All Fields] AND mediated[All Fields] AND ("dilatation"[MeSH Terms] OR "dilatation"[All Fields] OR "dilation"[All Fields])) AND ("hiv"[MeSH Terms] OR "hiv"[All Fields]) AND ("africa"[MeSH Terms] OR "africa"[All Fields])
