# Supplementary material for: The association between HIV and atherosclerotic cardiovascular disease in sub-Saharan Africa: a systematic review
Source: BMC Public Health. 2017 Dec 15;17:954. doi: 10.1186/s12889-017-4940-1 (PMC5732372; doi:10.1186/s12889-017-4940-1)
Supplement: Supplementary file 2 — Additional References. The following references primarily focused on CVD risk factors (n = 123) and biomarkers of immune status and endothelial activation (n = 12). These publications were assessed for eligibility but were excluded from the final qualitative analysis. (PDF 63 kb) [file 12889_2017_4940_MOESM2_ESM.pdf]

ADDITIONAL FILE 2

Additional References

CVD risk factors

1. Danwe C, Atchou G, Nkam M, Mbuagbaw J, Mougnoutou R, Nkouanfack C, et al. Effect of Antiretroviral Therapy on Lipid Metabolism in HIV/AIDS Subjects in Cameroon. J. Med. Sci. 2005;5(2):87-2.
2. Mutimura E, Stewart A, Rheeder P, Crowther NJ. Metabolic function and the prevalence of lipodystrophy in a population of HIV-infected African subjects receiving highly active antiretroviral therapy. J Acquir Immune Defic Syndr. 2007;46(4):451-5.
3. van Griensven J, De Naeyer L, Mushi T, Ubarijoro S, Gashumba D, Gazille C, et al. High prevalence of lipoatrophy among patients on stavudine-containing first-line antiretroviral therapy regimens in Rwanda. Trans R Soc Trop Med Hyg. 2007;101(8):793-8.
4. Barnighausen T, Welz T, Hosegood V, Batzing-Feigenbaum J, Tanser F, Herbst K, et al. Hiding in the shadows of the HIV epidemic: obesity and hypertension in a rural population with very high HIV prevalence in South Africa. J Hum Hypertens. 2008;22(3):236-9.
5. Buchacz K, Weidle PJ, Moore D, Were W, Mermin J, Downing R, et al. Changes in lipid profile over 24 months among adults on first-line highly active antiretroviral therapy in the home-based AIDS care program in rural Uganda. J Acquir Immune Defic Syndr. 2008;47(3):304-11.
6. Manuthu EM, Joshi MD, Lule GN, Karari E. Prevalence of dyslipidemia and dysglycaemia in HIV infected patients. East Afr Med J. 2008;85(1):10-7.
7. Mutimura E, Crowther NJ, Cade TW, Yarasheski KE, Stewart A. Exercise training reduces central adiposity and improves metabolic indices in HAART-treated HIV-positive

subjects in Rwanda: a randomized controlled trial. AIDS Res Hum Retroviruses. 2008;24(1):15-23.

8. Phillips AN, Carr A, Neuhaus J, Visnegarwala F, Prineas R, Burman WJ, et al. Interruption of antiretroviral therapy and risk of cardiovascular disease in persons with HIV-1 infection: exploratory analyses from the SMART trial. Antivir Ther. 2008;13(2):177-87.

9. Desalu OO, Oluboyo PO, Olokoba AB, Adekoya AO, Danburam A, Salawu FK, et al. Prevalence and determinants of tobacco smoking among HIV patients in North Eastern Nigeria. Afr J Med Med Sci. 2009;38(2):103-8.

10. George JA, Venter WD, Van Deventer HE, Crowther NJ. A longitudinal study of the changes in body fat and metabolic parameters in a South African population of HIV-positive patients receiving an antiretroviral therapeutic regimen containing stavudine. AIDS Res Hum Retroviruses. 2009;25(8):771-81.

11. Hattingh Z, Walsh C, Velman FJ, Bester CJ. The metabolic profiles of HIV-infected and non-infected women in Mangaung, South Africa. South Afr J Clin Nutr. 2009;22(1):23-8.

12. Jaquet A, Ekouevi DK, Aboubakrine M, Bashi J, Messou E, Maiga M, et al. Tobacco use and its determinants in HIV-infected patients on antiretroviral therapy in West African countries. Int J Tuberc Lung Dis. 2009;13(11):1433-9.

13. Lesi OA, Soyebi KS, Eboh CN. Fatty liver and hyperlipidemia in a cohort of HIV-positive Africans on highly active antiretroviral therapy. J Natl Med Assoc. 2009;101(2):151-5.

14. Mercier S, Gueye NF, Cournil A, Fontbonne A, Copin N, Ndiaye I, et al. Lipodystrophy and metabolic disorders in HIV-1-infected adults on 4- to 9-year antiretroviral therapy in Senegal: a case-control study. J Acquir Immune Defic Syndr. 2009;51(2):224-30.

15. Salami AK, Akande AA, Olokoba AB. Serum lipids and glucose abnormalities in HIV/AIDS patients on antiretroviral therapies. West Afr J Med. 2009;28(1):10-5.

16. Zannou DM, Denoeud L, Lacombe K, Amoussou-Guenou D, Bashi J, Akakpo J, et al. Incidence of lipodystrophy and metabolic disorders in patients starting non-nucleoside reverse transcriptase inhibitors in Benin. Antivir Ther. 2009;14(3):371-80.

17. Adewole OO, Eze S, Betiku Y, Anteyi E, Wada I, Ajuwon Z, et al. Lipid profile in HIV/AIDS patients in Nigeria. Afr Health Sci. 2010;10(2):144-9.

18. Anastos K, Ndamage F, Lu D, Cohen MH, Shi Q, Lazar J, et al. Lipoprotein levels and cardiovascular risk in HIV-infected and uninfected Rwandan women. AIDS Res Ther. 2010;7:34.

19. Fourie CM, Van Rooyen JM, Kruger A, Schutte AE. Lipid abnormalities in a never-treated HIV-1 subtype C-infected African population. Lipids. 2010;45(1):73-80.

20. Nguemaim NF, Mbuagbaw J, Nkoa T, Alemnji G, Teto G, Fanhi TC, et al. Serum lipid profile in highly active antiretroviral therapy-naive HIV-infected patients in Cameroon: a case-control study. HIV Med. 2010;11(6):353-9.

21. Armstrong C, Liu E, Okuma J, Spiegelman D, Guerino C, Njelekela M, et al. Dyslipidemia in an HIV-positive antiretroviral treatment-naive population in Dar es Salaam, Tanzania. J Acquir Immune Defic Syndr. 2011;57(2):141-5.

22. Bloomfield GS, Hogan JW, Keter A, Sang E, Carter EJ, Velazquez EJ, et al. Hypertension and obesity as cardiovascular risk factors among HIV seropositive patients in Western Kenya. PLoS One. 2011;6(7):e22288.

23. Dave JA, Lambert EV, Badri M, West S, Maartens G, Levitt NS. Effect of nonnucleoside reverse transcriptase inhibitor-based antiretroviral therapy on dysglycemia and insulin sensitivity in South African HIV-infected patients. J Acquir Immune Defic Syndr. 2011;57(4):284-9.

24. Fontbonne A, Cournil A, Cames C, Mercier S, Coly AN, Lacroux A, et al. Anthropometric characteristics and cardiometabolic risk factors in a sample of urban-dwelling adults in Senegal. Diabetes Metab. 2011;37(1):52-8.

25. Julius H, Basu D, Ricci E, Wing J, Basu JK, Pocaterra D, et al. The burden of metabolic diseases amongst HIV positive patients on HAART attending The Johannesburg Hospital. Curr HIV Res. 2011;9(4):247-52.

26. Muronya W, Sanga E, Talama G, Kumwenda JJ, van Oosterhout JJ. Cardiovascular risk factors in adult Malawians on long-term antiretroviral therapy. Trans R Soc Trop Med Hyg. 2011;105(11):644-9.

27. Pefura Yone EW, Betyoumin AF, Kengne AP, Kaze Folefack FJ, Ngogang J. First-line antiretroviral therapy and dyslipidemia in people living with HIV-1 in Cameroon: a cross-sectional study. AIDS Res Ther. 2011;8:33.

28. Shapiro AE, Tshabangu N, Golub JE, Martinson NA. Intention to quit smoking among human immunodeficiency virus infected adults in Johannesburg, South Africa. Int J Tuberc Lung Dis. 2011;15(1):140-2.

29. Ayodele OE, Akinboro AO, Akinyemi SO, Adepeju AA, Akinremi OA, Alao CA, et al. Prevalence and clinical correlates of metabolic syndrome in Nigerians living with human immunodeficiency virus/acquired immunodeficiency syndrome. Metab Syndr Relat Disord. 2012;10(5):373-9.

30. Berhane T, Yami A, Alemseged F, Yemane T, Hamza L, Kassim M, et al. Prevalence of lipodystrophy and metabolic syndrome among HIV positive individuals on Highly Active Anti-Retroviral treatment in Jimma, South West Ethiopia. Pan Afr Med J. 2012;13:43.

31. Chamie G, Kwarisiima D, Clark TD, Kabami J, Jain V, Geng E, et al. Leveraging rapid community-based HIV testing campaigns for non-communicable diseases in rural Uganda. PLoS One. 2012;7(8):e43400.

32. Chillo P, Bakari M, Lwakatare J. Echocardiographic diagnoses in HIV-infected patients presenting with cardiac symptoms at Muhimbili National Hospital in Dar es Salaam, Tanzania. Cardiovasc J Afr. 2012;23(2):90-7.

33. Feleke Y, Fekade D, Mezegebu Y. Prevalence of highly active antiretroviral therapy associated metabolic abnormalities and lipodystrophy in HIV infected patients. Ethiop Med J. 2012;50(3):221-30.

34. Malaza A, Mossong J, Barnighausen T, Newell ML. Hypertension and obesity in adults living in a high HIV prevalence rural area in South Africa. PLoS One. 2012;7(10):e47761.

35. Mandina Ndona M, Longo-Mbenza B, Wumba R, Tandu Umba B, Buassa-Bu-Tsumbu B, Mbula Mambimbi M, et al. Nadir CD4+, religion, antiretroviral therapy, incidence of type 2 diabetes mellitus, and increasing rates of obesity among black Africans with HIV disease. Int J Gen Med. 2012;5:983-90.

36. Negin J, Martiniuk A, Cumming RG, Naidoo N, Phaswana-Mafuya N, Madurai L, et al. Prevalence of HIV and chronic comorbidities among older adults. AIDS. 2012;26 Suppl 1:S55-63.

37. Omech B, Sempa J, Castelnuovo B, Opio K, Otim M, Mayanja-Kizza H, et al. Prevalence of HIV-associated metabolic abnormalities among patients taking first-line antiretroviral therapy in Uganda. ISRN AIDS. 2012;2012:960178.

38. Oni T, Gideon HP, Bangani N, Tsekela R, Seldon R, Wood K, et al. Smoking, BCG and employment and the risk of tuberculosis infection in HIV-infected persons in South Africa. PLoS One. 2012;7(10):e47072.

39. Pefura Yone EW, Kengne AP, Ashuntantang G, Betyoumin AF, Ngogang J. Dyslipidaemia in HIV-1-infected patients receiving protease inhibitors after initial treatment with first-line-based non-nucleoside reverse transcriptase inhibitors: a cross-sectional study. BMJ Open. 2012;2(4).

40. Zhou J, Lurie MN, Barnighausen T, McGarvey ST, Newell ML, Tanser F. Determinants and spatial patterns of adult overweight and hypertension in a high HIV prevalence rural South African population. Health Place. 2012;18(6):1300-6.

41. Bakari M, Chillo P, Lwakatare J. Factors associated with, and echocardiographic findings of heart failure among HIV infected patients at a tertiary health care facility in Dar es Salaam, Tanzania. Tanzan J Health Res. 2013;15(2):73-81.

42. Daniyam C, Iroezindu M. Lipid Profile of Anti-Retroviral Treatment-Naive HIV-Infected Patients in Jos, Nigeria. Ann Med Health Sci Res. 2013;3(1):26-30.

43. Edward AO, Oladayo AA, Omolola AS, Adetiloye AA, Adedayo PA. Prevalence of traditional cardiovascular risk factors and evaluation of cardiovascular risk using three risk equations in nigerians living with human immunodeficiency virus. N Am J Med Sci. 2013;5(12):680-8.

44. Ekali LG, Johnstone LK, Echouffo-Tcheugui JB, Kouanfack C, Dehayem MY, Fezeu L, et al. Fasting blood glucose and insulin sensitivity are unaffected by HAART duration in Cameroonians receiving first-line antiretroviral treatment. Diabetes Metab. 2013;39(1):71-7.

45. Frantz JM, Murenzi A. The physical activity levels among people living with human immunodeficiency virus/acquired immunodeficiency syndrome receiving high active antiretroviral therapy in Rwanda. SAHARA J. 2013;10(3-4):113-8.

46. Goedecke JH, Micklesfield LK, Levitt NS, Lambert EV, West S, Maartens G, et al. Effect of different antiretroviral drug regimens on body fat distribution of HIV-infected South African women. AIDS Res Hum Retroviruses. 2013;29(3):557-63.

47. Govindasamy D, Kranzer K, van Schaik N, Noubary F, Wood R, Walensky RP, et al. Linkage to HIV, TB and non-communicable disease care from a mobile testing unit in Cape Town, South Africa. PLoS One. 2013;8(11):e80017.

48. Kiage JN, Heimburger DC, Nyirenda CK, Wellons MF, Bagchi S, Chi BH, et al. Cardiometabolic risk factors among HIV patients on antiretroviral therapy. Lipids Health Dis. 2013;12:50.

49. Lam C, Martinson N, Hepp L, Ambrose B, Msandiwa R, Wong ML, et al. Prevalence of tobacco smoking in adults with tuberculosis in South Africa. Int J Tuberc Lung Dis. 2013;17(10):1354-7.

50. Liu E, Armstrong C, Spiegelman D, Chalamilla G, Njelekela M, Hawkins C, et al. First-line antiretroviral therapy and changes in lipid levels over 3 years among HIV-infected adults in Tanzania. Clin Infect Dis. 2013;56(12):1820-8.

51. Mateen FJ, Kanters S, Kalyesubula R, Mukasa B, Kawuma E, Kengne AP, et al. Hypertension prevalence and Framingham risk score stratification in a large HIV-positive cohort in Uganda. J Hypertens. 2013;31(7):1372-8; discussion 8.

52. Moyo D, Tanthuma G, Mushisha O, Kwadiba G, Chikuse F, Cary MS, et al. Diabetes mellitus in HIV-infected patients receiving antiretroviral therapy. S Afr Med J. 2013;104(1):37-9.

53. Muhammad S, Sani MU, Okeahialam BN. Prevalence of dyslipidemia among human immunodeficiency virus infected Nigerians. Ann Afr Med. 2013;12(1):24-8.

54. Muhammad S, Sani MU, Okeahialam BN. Cardiovascular disease risk factors among HIV-infected Nigerians receiving highly active antiretroviral therapy. Niger Med J. 2013;54(3):185-90.

55. Ngala RA, Fianko K. Dyslipidaemia and dysglycaemia in HIV-infected patients on highly active anti-retroviral therapy in Kumasi Metropolis. Afr Health Sci. 2013;13(4):1107-16.

56. Parikh SM, Obuku EA, Walker SA, Semeere AS, Auerbach BJ, Hakim JG, et al. Clinical differences between younger and older adults with HIV/AIDS starting antiretroviral therapy in Uganda and Zimbabwe: a secondary analysis of the DART trial. PLoS One. 2013;8(10):e76158.

57. Sinxadi PZ, Dave JA, Samuels DC, Heckmann JM, Maartens G, Levitt NS, et al. Mitochondrial genomics and antiretroviral therapy-associated metabolic complications in HIV-infected Black South Africans: a pilot study. AIDS Res Hum Retroviruses. 2013;29(7):1031-9.

58. Waweru P, Anderson R, Steel H, Venter WD, Murdoch D, Feldman C. The prevalence of smoking and the knowledge of smoking hazards and smoking cessation strategies among HIV-positive patients in Johannesburg, South Africa. S Afr Med J. 2013;103(11):858-60.

59. Abebe M, Kinde S, Belay G, Gebreegziabxier A, Challa F, Gebeyehu T, et al. Antiretroviral treatment associated hyperglycemia and dyslipidemia among HIV infected patients at Burayu Health Center, Addis Ababa, Ethiopia: a cross-sectional comparative study. BMC Res Notes. 2014;7:380.

60. Bekolo CE, Nguena MB, Ewane L, Bekoule PS, Kollo B. The lipid profile of HIV-infected patients receiving antiretroviral therapy in a rural Cameroonian population. BMC Public Health. 2014;14:236.

61. Bloomfield GS, Hogan JW, Keter A, Holland TL, Sang E, Kimaiyo S, et al. Blood pressure level impacts risk of death among HIV seropositive adults in Kenya: a retrospective analysis of electronic health records. BMC Infect Dis. 2014;14:284.

62. Borkum M, Wearne N, Alfred A, Dave JA, Levitt NS, Rayner B. Ambulatory blood pressure profiles in a subset of HIV-positive patients pre and post antiretroviral therapy. Cardiovasc J Afr. 2014;25(4):153-7.

63. Gomo ZA, Hakim JG, Walker SA, Tinago W, Mandozana G, Kityo C, et al. Impact of second-line antiretroviral regimens on lipid profiles in an African setting: the DART trial sub-study. AIDS Res Ther. 2014;11(1):32.

64. Guira O, Tieno H, Yameogo B, Diendere AE, Korsaga N, Sagna Y, et al. Characteristics and factors associated with the clinical forms of lipoatrophy during highly active antiretroviral therapy in Ouagadougou, Burkina Faso. J Int Assoc Provid AIDS Care. 2014;13(2):184-7.

65. Kagaruki GB, Mayige MT, Ngadaya ES, Kimaro GD, Kalinga AK, Kilale AM, et al. Magnitude and risk factors of non-communicable diseases among people living with HIV in Tanzania: a cross sectional study from Mbeya and Dar es Salaam regions. BMC Public Health. 2014;14:904.

66. Menezes CN, Crowther NJ, Duarte R, Van Amsterdam D, Evans D, Dickens C, et al. A randomized clinical trial comparing metabolic parameters after 48 weeks of standard- and low-dose stavudine therapy and tenofovir disoproxil fumarate therapy in HIV-infected South African patients. HIV Med. 2014;15(1):3-12.

67. Ngo-Matip ME, Pieme CA, Azabji-Kenfack M, Biapa PC, Germaine N, Heike E, et al. Effects of Spirulina platensis supplementation on lipid profile in HIV-infected antiretroviral naive patients in Yaounde-Cameroon: a randomized trial study. Lipids Health Dis. 2014;13:191.

68. Ogbera AO, Kapur A, Odeyemi K, Longe-Peters K, Adeyeye OO, Odeniyi I, et al. Screening for diabetes mellitus and human immunodefiency virus infection in persons with tuberculosis. J Prev Med Hyg. 2014;55(2):42-5.

69. Ogunmola OJ, Oladosu OY, Olamoyegun AM. Association of hypertension and obesity with HIV and antiretroviral therapy in a rural tertiary health center in Nigeria: a cross-sectional cohort study. Vasc Health Risk Manag. 2014;10:129-37.

70. Peck RN, Shedafa R, Kalluvya S, Downs JA, Todd J, Suthanthiran M, et al. Hypertension, kidney disease, HIV and antiretroviral therapy among Tanzanian adults: a cross-sectional study. BMC Med. 2014;12:125.

71. Shaffer D, Hughes MD, Sawe F, Bao Y, Moses A, Hogg E, et al. Cardiovascular disease risk factors in HIV-infected women after initiation of lopinavir/ritonavir- and nevirapine-based antiretroviral therapy in Sub-Saharan Africa: A5208 (OCTANE). J Acquir Immune Defic Syndr. 2014;66(2):155-63.

72. Tesfaye DY, Kinde S, Medhin G, Megerssa YC, Tadewos A, Tadesse E, et al. Burden of metabolic syndrome among HIV-infected patients in Southern Ethiopia. Diabetes Metab Syndr. 2014;8(2):102-7.

73. van Rooyen JM, Fourie CM, Steyn HS, Koekemoer G, Huisman HW, Schutte R, et al. Cardiometabolic markers to identify cardiovascular disease risk in HIV-infected black South Africans. S Afr Med J. 2014;104(3):195-9.

74. Wrottesley SV, Micklesfield LK, Hamill MM, Goldberg GR, Prentice A, Pettifor JM, et al. Dietary intake and body composition in HIV-positive and -negative South African women. Public Health Nutr. 2014;17(7):1603-13.

75. Abrahams Z, Dave JA, Maartens G, Levitt NS. Changes in blood pressure, glucose levels, insulin secretion and anthropometry after long term exposure to antiretroviral therapy in South African women. AIDS Res Ther. 2015;12:24.

76. Asiki G, Murphy GA, Baisley K, Nsubuga RN, Karabarinde A, Newton R, et al. Prevalence of dyslipidaemia and associated risk factors in a rural population in South-Western Uganda: a community based survey. PLoS One. 2015;10(5):e0126166.

77. Clark SJ, Gomez-Olive FX, Houle B, Thorogood M, Klipstein-Grobusch K, Angotti N, et al. Cardiometabolic disease risk and HIV status in rural South Africa: establishing a baseline. BMC Public Health. 2015;15:135.

78. Edwards JK, Bygrave H, Van den Bergh R, Kizito W, Cheti E, Kosgei RJ, et al. HIV with non-communicable diseases in primary care in Kibera, Nairobi, Kenya: characteristics and outcomes 2010-2013. Trans R Soc Trop Med Hyg. 2015;109(7):440-6.

79. Eholie SP, Lacombe K, Krain A, Diallo Z, Ouiminga M, Campa P, et al. Metabolic disorders and cardiovascular risk in treatment-naive HIV-infected patients of sub-saharan origin starting antiretrovirals: impact of westernized lifestyle. AIDS Res Hum Retroviruses. 2015;31(4):384-92.

80. Erlandson KM, Taejaroenkul S, Smeaton L, Gupta A, Singini IL, Lama JR, et al. A Randomized Comparison of Anthropomorphic Changes With Preferred and Alternative Efavirenz-Based Antiretroviral Regimens in Diverse Multinational Settings. Open Forum Infect Dis. 2015;2(3):ofv095.

81. Kavishe B, Biraro S, Baisley K, Vanobberghen F, Kapiga S, Munderi P, et al. High prevalence of hypertension and of risk factors for non-communicable diseases (NCDs): a population based cross-sectional survey of NCDS and HIV infection in Northwestern Tanzania and Southern Uganda. BMC Med. 2015;13:126.

82. Kuti MA, Adesina OA, Awolude OA, Ogunbosi BO, Fayemiwo SA, Akinyemi JO, et al. Dyslipidemia in ART-Naive HIV-Infected Persons in Nigeria--Implications for Care. J Int Assoc Provid AIDS Care. 2015;14(4):355-9.

83. Maganga E, Smart LR, Kalluvya S, Kataraihya JB, Saleh AM, Obeid L, et al. Glucose Metabolism Disorders, HIV and Antiretroviral Therapy among Tanzanian Adults. PLoS One. 2015;10(8):e0134410.

84. Mashinya F, Alberts M, Van Geertruyden JP, Colebunders R. Assessment of cardiovascular risk factors in people with HIV infection treated with ART in rural South Africa: a cross sectional study. AIDS Res Ther. 2015;12:42.

85. Menanga AP, Ngomseu CK, Jingi AM, Mfangam BM, Noubiap JJ, Gweth MN, et al. Patterns of cardiovascular disease in a group of HIV-infected adults in Yaounde, Cameroon. Cardiovasc Diagn Ther. 2015;5(6):420-7.

86. Myerson M. Lipid management in human immunodeficiency virus. Cardiol Clin. 2015;33(2):277-98.

87. Nakibuuka J, Sajatovic M, Nankabirwa J, Furlan AJ, Kayima J, Ddumba E, et al. Stroke-Risk Factors Differ between Rural and Urban Communities: Population Survey in Central Uganda. Neuroepidemiology. 2015;44(3):156-65.

88. Nsagha DS, Weledji EP, Assob NJ, Njunda LA, Tanue EA, Kibu OD, et al. Highly active antiretroviral therapy and dyslipidemia in people living with HIV/AIDS in Fako Division, South West Region of Cameroon. BMC Cardiovasc Disord. 2015;15:95.

89. Okello S, Kanyesigye M, Muyindike WR, Annex BH, Hunt PW, Haneuse S, et al. Incidence and predictors of hypertension in adults with HIV-initiating antiretroviral therapy in south-western Uganda. J Hypertens. 2015;33(10):2039-45.

90. Oni T, Youngblood E, Boulle A, McGrath N, Wilkinson RJ, Levitt NS. Patterns of HIV, TB, and non-communicable disease multi-morbidity in peri-urban South Africa- a cross sectional study. BMC Infect Dis. 2015;15:20.

91. Rabkin M, Mutiti A, Chung C, Zhang Y, Wei Y, El-Sadr WM. Missed opportunities to address cardiovascular disease risk factors amongst adults attending an urban HIV clinic in South Africa. PLoS One. 2015;10(10):e0140298.

92. Sander LD, Newell K, Ssebbowa P, Serwadda D, Quinn TC, Gray RH, et al. Hypertension, cardiovascular risk factors and antihypertensive medication utilisation among HIV-infected individuals in Rakai, Uganda. Trop Med Int Health. 2015;20(3):391-6.

93. Soliman EZ, Sharma S, Arasteh K, Wohl D, Achhra A, Tambussi G, et al. Baseline cardiovascular risk in the INSIGHT Strategic Timing of AntiRetroviral Treatment (START) trial. HIV Med. 2015;16 Suppl 1:46-54.

94. Wensink GE, Schoffelen AF, Tempelman HA, Rookmaaker MB, Hoepelman AI, Barth RE. Albuminuria Is Associated with Traditional Cardiovascular Risk Factors and Viral Load in HIV-Infected Patients in Rural South Africa. PLoS One. 2015;10(8):e0136529.

95. Zhou DT, Kodogo V, Chokuona KF, Gomo E, Oektedalen O, Stray-Pedersen B. Dyslipidemia and cardiovascular disease risk profiles of patients attending an HIV treatment clinic in Harare, Zimbabwe. HIV AIDS (Auckl). 2015;7:145-55.

96. Dave JA, Levitt NS, Ross IL, Lacerda M, Maartens G, Blom D. Anti-Retroviral Therapy Increases the Prevalence of Dyslipidemia in South African HIV-Infected Patients. PLoS One. 2016;11(3):e0151911.

97. Dimala CA, Atashili J, Mbuagbaw JC, Wilfred A, Monekosso GL. Prevalence of Hypertension in HIV/AIDS Patients on Highly Active Antiretroviral Therapy (HAART) Compared with HAART-Naive Patients at the Limbe Regional Hospital, Cameroon. PLoS One. 2016;11(2):e0148100.

98. Guira O, Tieno H, Diendere AE, Sagna Y, Diallo I, Yameogo B, et al. Features of Metabolic Syndrome and Its Associated Factors during Highly Active Antiretroviral Therapy in Ouagadougou (Burkina Faso). J Int Assoc Provid AIDS Care. 2016;15(2):159-63.

99. Hirigo AT, Tesfaye DY. Influences of gender in metabolic syndrome and its components among people living with HIV virus using antiretroviral treatment in Hawassa, southern Ethiopia. BMC Res Notes. 2016;9:145.

100. Karamchand S, Leisegang R, Schomaker M, Maartens G, Walters L, Hislop M, et al. Risk Factors for Incident Diabetes in a Cohort Taking First-Line Nonnucleoside Reverse Transcriptase Inhibitor-Based Antiretroviral Therapy. Medicine (Baltimore). 2016;95(9):e2844.

101. Kwarisiima D, Balzer L, Heller D, Kotwani P, Chamie G, Clark T, et al. Population-Based Assessment of Hypertension Epidemiology and Risk Factors among HIV-Positive and General Populations in Rural Uganda. PLoS One. 2016;11(5):e0156309.

102. Levitt NS, Peer N, Steyn K, Lombard C, Maartens G, Lambert EV, et al. Increased risk of dysglycaemia in South Africans with HIV; especially those on protease inhibitors. Diabetes Res Clin Pract. 2016;119:41-7.

103. Magodoro IM, Esterhuizen TM, Chivese T. A cross-sectional, facility based study of comorbid non-communicable diseases among adults living with HIV infection in Zimbabwe. BMC Res Notes. 2016;9:379.

104. Mashinya F, Alberts M, Colebunders R, Van Geertruyden JP. Weight status and associated factors among HIV infected people on antiretroviral therapy in rural Dikgale, Limpopo, South Africa. Afr J Prim Health Care Fam Med. 2016;8(1):e1-e8.

105. Matoga MM, Hosseinipour MC, Aga E, Ribaudo HJ, Kumarasamy N, Bartlett J, et al. Hyperlipidaemia in HIV-infected patients on lopinavir/ritonavir monotherapy in resource-limited settings. Antivir Ther. 2016.

106. Mugisha JO, Schatz EJ, Randell M, Kuteesa M, Kowal P, Negin J, et al. Chronic disease, risk factors and disability in adults aged 50 and above living with and without HIV: findings from the Wellbeing of Older People Study in Uganda. Glob Health Action. 2016;9:31098.

107. Muyanja D, Muzoora C, Muyingo A, Muyindike W, Siedner MJ. High prevalence of metabolic syndrome and cardiovascular disease risk among people with HIV on stable ART in southwestern Uganda. AIDS Patient Care STDS. 2016;30(1):4-10.

108. Nduka CU, Stranges S, Bloomfield GS, Kimani PK, Achinge G, Malu AO, et al. A plausible causal link between antiretroviral therapy and increased blood pressure in a sub-Saharan African setting: A propensity score-matched analysis. Int J Cardiol. 2016;220:400-7.

109. Nduka CU, Uthman OA, Kimani PK, Malu AO, Stranges S. Impact of body fat changes in mediating the effects of antiretroviral therapy on blood pressure in HIV-infected persons in a sub-Saharan African setting. Infect Dis Poverty. 2016;5(1):55.

110. Njelekela M, Muhihi A, Aveika A, Spiegelman D, Hawkins C, Armstrong C, et al. Prevalence of hypertension and its associated risk factors among 34,111 HAART naive HIV-infected adults in Dar es Salaam, Tanzania. Int J Hypertens. 2016;2016:5958382.

111. Ombeni W, Kamuhabwa AR. Lipid profile in HIV-infected patients using first-line antiretroviral drugs. J Int Assoc Provid AIDS Care. 2016;15(2):164-71.

112. Osegbe ID, Soriyan OO, Ogbenna AA, Okpara HC, Azinge EC. Risk factors and assessment for cardiovascular disease among HIV-positive patients attending a Nigerian tertiary hospital. Pan Afr Med J. 2016;23:206.

113. Pillay S, Aldous C, Mahomed F. A deadly combination - HIV and diabetes mellitus: Where are we now? S Afr Med J. 2016;106(4):54.

114. Rhee JY, Bahtila TD, Palmer D, Tih PM, Aberg JA, LeRoith D, et al. Prediabetes and diabetes among HIV-infected adults in Cameroon. Diabetes Metab Res Rev. 2016;32(6):544-9.

115. Sinxadi PZ, McIlleron HM, Dave JA, Smith PJ, Levitt NS, Haas DW, et al. Plasma efavirenz concentrations are associated with lipid and glucose concentrations. Medicine (Baltimore). 2016;95(2):e2385.

116. Sobieszczyk ME, Werner L, Mlisana K, Naicker N, Feinstein A, Gray CM, et al. Metabolic syndrome after HIV acquisition in South African women. J Acquir Immune Defic Syndr. 2016;73(4):438-45.

117. Steiniche D, Jespersen S, Erikstrup C, Krarup H, Handberg A, Ostergaard L, et al. Diabetes mellitus and impaired fasting glucose in ART-naive patients with HIV-1, HIV-2 and HIV-1/2 dual infection in Guinea-Bissau: a cross-sectional study. Trans R Soc Trop Med Hyg. 2016;110(4):219-27.

118. Zhou DT, Nehumba D, Oktedalen O, Marange P, Kodogo V, Gomo ZA, et al. Changes in lipid profiles of HIV(+) adults over nine months at a Harare HIV Clinic: a longitudinal study. Biochem Res Int. 2016;2016:3204818.

119. Zhou DT, Oektedalen O, Shawarira-Bote S, Stray-Pedersen B. Changes in coronary heart disease risk profiles of HIV patients in Zimbabwe over 9 months: a follow-up study. HIV AIDS (Auckl). 2016;8:165-74.

120. Gaziano TA, Abrahams-Gessel S, Gomez-Olive FX, Wade A, Crowther NJ, Alam S, et al. Cardiometabolic risk in a population of older adults with multiple co-morbidities in rural south africa: the HAALSI (Health and Aging in Africa: longitudinal studies of INDEPTH communities) study. BMC Public Health. 2017;17(1):206.

121. Nlooto M. Comorbidities of HIV infection and health care seeking behavior among HIV infected patients attending public sector healthcare facilities in KwaZulu-Natal: A cross sectional study. PLoS One. 2017;12(2):e0170983.

122. PrayGod G, Changalucha J, Kapiga S, Peck R, Todd J, Filteau S. Dysglycemia associations with adipose tissue among HIV-infected patients after 2 years of antiretroviral therapy in Mwanza: a follow-up cross-sectional study. BMC Infect Dis. 2017;17(1):103.

123. Rodriguez-Arboli E, Mwamelo K, Kalinjuma AV, Furrer H, Hatz C, Tanner M, et al. Incidence and risk factors for hypertension among HIV patients in rural Tanzania - A prospective cohort study. PLoS One. 2017;12(3):e0172089.

Biomarkers of immune status and endothelial activation

1. Haissman JM, Vestergaard LS, Sembuche S, Erikstrup C, Mmbando B, Mtullu S, et al. Plasma cytokine levels in Tanzanian HIV-1-infected adults and the effect of antiretroviral treatment. J Acquir Immune Defic Syndr. 2009;52(4):493-7.

2. Mayne E, Funderburg NT, Sieg SF, Asaad R, Kalinowska M, Rodriguez B, et al. Increased platelet and microparticle activation in HIV infection: upregulation of P-selectin and tissue factor expression. J Acquir Immune Defic Syndr. 2012;59(4):340-6.

3. Glyn MC, Van Rooyen JM, Schutte R, Huisman HW, Boger RH, Schwedhelm E, et al. A comparison of the association between glomerular filtration and L-arginine status in HIV-infected and uninfected African men: the SAfrEIC study. J Hum Hypertens. 2013;27(9):557-63.

4. Graham SM, Rajwans N, Jaoko W, Estambale BB, McClelland RS, Overbaugh J, et al. Endothelial activation biomarkers increase after HIV-1 acquisition: plasma vascular cell adhesion molecule-1 predicts disease progression. AIDS. 2013;27(11):1803-13.

5. Graham SM, Rajwans N, Tapia KA, Jaoko W, Estambale BB, McClelland RS, et al. A prospective study of endothelial activation biomarkers, including plasma angiopoietin-1 and angiopoietin-2, in Kenyan women initiating antiretroviral therapy. BMC Infect Dis. 2013;13:263.

6. Mutevedzi PC, Rodger AJ, Kowal P, Nyirenda M, Newell ML. Decreased chronic morbidity but elevated HIV associated cytokine levels in HIV-infected older adults receiving HIV treatment: benefit of enhanced access to care? PLoS One. 2013;8(10):e77379.

7. Hudson CL, Zemlin AE, Ipp H. The cardiovascular risk marker asymmetric dimethylarginine is elevated in asymptomatic, untreated HIV-1 infection and correlates with markers of immune activation and disease progression. Ann Clin Biochem. 2014;51(Pt 5):568-75.

8. Bestawros M, Chidumayo T, Blevins M, Canipe A, Bala J, Kelly P, et al. Increased systemic inflammation is associated with cardiac and vascular dysfunction over the first 12 weeks of antiretroviral therapy among undernourished, HIV-infected adults in Southern Africa. J AIDS Clin Res. 2015;6(3).

9. Nkambule BB, Davison GM, Ipp H. The evaluation of platelet indices and markers of inflammation, coagulation and disease progression in treatment-naive, asymptomatic HIV-infected individuals. Int J Lab Hematol. 2015;37(4):450-8.

10. Gori E, Mduluza T, Nyagura M, Stray-Pedersen B, Gomo ZA. Inflammation-modulating cytokine profile and lipid interaction in HIV-related risk factors for cardiovascular diseases. Ther Clin Risk Manag. 2016;12:1659-66.

11. Siedner MJ, Kim JH, Nakku RS, Bibangambah P, Hemphill L, Triant VA, et al. Persistent immune activation and carotid atherosclerosis in HIV-infected Ugandans receiving antiretroviral therapy. J Infect Dis. 2016;213(3):370-8.

12. Janssen S, Huson MA, Osbak KK, Rossatanga EG, Alabi A, Lutter R, et al. HIV infection rather than concurrent opportunistic infections drives most systemic procoagulant, vascular and damage responses - a prospective cohort study in central Africa. Antivir Ther. 2017;22(2):153-61.
